# A Multifunctional Chitosan-Coated Ciprofloxacin–Luteolin Lipid Nanoplatform for Photodynamic Therapy of Prostatic Cancer

**DOI:** 10.3390/ijms27177594

**Published:** 2026-08-25

**Authors:** Riyad F. Alzhrani, Khalid Alamer, Nasser Alothaymin, Rabbani Syed, Ahmed H. Bakheit, Gamaleldin I. Harisa

**Affiliations:** 1 Department of Pharmaceutics, College of Pharmacy, King Saud University, Riyadh 11451, Saudi Arabia; rfalzahrani@ksu.edu.sa (R.F.A.); khalidalamer1949@gmail.com (K.A.); nasseralothaymin@gmail.com (N.A.); rsyed@ksu.edu.sa (R.S.); 2 Department of Pharmaceutical Chemistry, College of Pharmacy, King Saud University, Riyadh 11451, Saudi Arabia; abujazz76@gmail.com

**Keywords:** prostate cancer, photodynamic therapy, chitosan-coated lipid nanoparticles, ciprofloxacin, luteolin, drug co-delivery, reactive oxygen species, molecular docking, nanomedicine

## Abstract

This study developed chitosan (CS)-coated lipid nanoparticles (LNPs) co-loaded with CIP and LUT (CCIPLUTLNPs) as a multifunctional photodynamic therapy (PDT)-enhanced nanoplatform for PCA therapy. The formulations were characterized by particle size, polydispersity index, zeta potential, and encapsulation efficiency. Stability and hemocompatibility were also evaluated. Anticancer activity was assessed in PC-3 cells by measuring cell viability, intracellular reactive oxygen species (ROS) generation under dark and sunlight irradiation, and mitochondrial membrane potential (MMP). In addition, molecular docking was performed against 5α-reductase, androgen receptor (AR), and DNA topoisomerase IIα (TOP2A). Results: The developed LNPs exhibited nanoscale particle size, narrow size distribution, high drug encapsulation efficiency, and a positive surface charge following CS coating. The CS-coated LNPs demonstrated good storage stability and excellent hemocompatibility. Moreover, CCIPLUTLNPs produced the greatest cytotoxicity, enhanced ROS generation, and pronounced mitochondrial membrane depolarization in PC-3 cells. Sunlight irradiation significantly increased ROS production compared with dark conditions, confirming enhanced PDT activity. Molecular docking revealed favorable binding of CIP and LUT to 5α-reductase, AR, and TOP2A, supporting a multitarget anticancer mechanism. Conclusion: CCIPLUTLNPs represent a promising multifunctional nanoplatform that integrates efficient drug delivery, ROS-mediated PDT, and multitarget molecular interactions, offering a potential strategy for improved PCA treatment.

## 1. Introduction

Prostate cancer (PCA) is one of the most frequently diagnosed malignancies and remains a leading cause of cancer-related mortality among men worldwide. Its incidence increases markedly with advancing age and is influenced by genetic predisposition, family history, ethnicity, and obesity. In addition, chronic inflammation, hormonal dysregulation, dietary habits, and environmental exposures have been recognized as key factors contributing to PCA development and progression [1]. Malignant transformation of prostate epithelial cells is a multistep process driven by genetic and epigenetic changes, resulting in dysregulation of androgen receptor (AR) signaling, activation of oncogenic pathways such as PI3K/AKT/mTOR and MAPK [2]. Additionally, loss of tumor suppressors genes, evasion of apoptosis, genomic instability, increased proliferative capacity, angiogenesis, and metastatic distribution are linked with PCA [3].

Despite advances in PCA treatment, disease progression and recurrence remain major challenges due to treatment resistance, AR reactivation, dysregulated survival pathways, enhanced DNA repair, and apoptosis evasion. Additionally, treatment-related systemic toxicity highlights the need for safer and more effective therapeutic strategies [4]. In this context, photodynamic therapy (PDT) is a promising minimally invasive and highly targeted therapeutic strategy for PCA treatment [5,6]. PDT relies on the activation of synthetic or natural photosensitizers by light to generate reactive oxygen species (ROS) that selectively kill tumor cells in a selective manner. The combination of PDT with photothermal therapy, photoimmunotherapy, chemotherapy, and other therapies improves PCA therapeutic outcomes [5]. The advanced photosensitizers improved diagnosis and enhanced treatment precision and clinical outcomes [6].

Drug repurposing is an attractive approach in cancer therapy because it uses approved or well-characterized compounds with established biological effects and safety profiles, thereby lowering the cost and time required for drug development [7]. For instance, Ciprofloxacin (CIP) is fluoroquinolone antibiotic, was repurposed as an anticancer agent. CIP inhibits topoisomerase II alpha (TOP2A) activity [8]. Thus, CIP prevents DNA replication, hinders cell division, and induced the death of cancer cells [8]. Moreover, CIP possesses intrinsic photosensitizing properties and, upon photoactivation with ultraviolet-A (UVA) irradiation, generates ROS that enhance cancer cell death. These photochemical characteristics may contribute to its selective anticancer activity and highlight its potential as a photosensitizer for PDT in PCA [9]. Despite this, CIP application in PCA remains limited due to the insufficient CIP concentrations achievable within prostate tissue. Therefore, drug combination strategies and nanomedicine approaches may enhance CIP therapeutic efficacy in PCA [10].

Additionally, luteolin (LUT) is a bioflavonoid present in many fruits, vegetables, and medicinal plants. LUT was reported as a multitargeted anticancer agent. For instance, LUT elicits cytotoxic effect in PCA by modifying PI3K/AKT/mTOR, MAPK, NF-κB, and AR signaling [11]. Therefore, LUT was suggested to suppress the proliferation and progression of cancer cells [11,12]. LUT augments the sensitivity of cancer cells to chemotherapeutic agents through triggering oxidative stress, apoptosis and ferroptosis in cancer cells [11,13]. Therefore, LUT is an attractive therapeutic candidate for the treatment of PCA. Nevertheless, LUT clinical translation remains limited due to poor aqueous solubility, low oral bioavailability, and fast metabolic degradation. These limitations underscore the need for effective drug-delivery plans to maximize LUT therapeutic outcomes [13,14].

Together, the combination of repurposed drugs with bioactive agents offers a hopeful plan for accomplishing synergistic anticancer effects [15,16,17]. Additionally, nanomedicine-based drug delivery systems have gained significant attention for overcoming the boundaries associated with classical therapy [18]. Lipid nanoparticles (LNPs) offer a biocompatible and scalable platform that protects drugs, improves bioavailability, prolongs circulation, and enhances tumor accumulation. Their ability to co-deliver multiple drugs may promote synergistic effects while reducing toxicity. Moreover, chitosan (CS) surface modification can further enhance the therapeutic performance of LNPs [18]. CS is a biodegradable, biocompatible, and mucoadhesive cationic polymer that generates a positive charge on LNPs’ exterior [4,18,19]. The cationic nature of CS-coated LNPs promotes electrostatic interactions with negatively charged biomembranes. Specifically, PCA cells frequently exhibit increased surface negativity due to aberrant hypersialylation of their membrane [20]. This negative glycocalyx can attract cationic nanocarriers, enhance cellular uptake, and improve intracellular drug delivery [4,20,21].

This study aimed to develop and characterize CS-coated LNPs co-loaded with CIP and LUT (CCIPLUTLNPs) for PDT-enhanced treatment of PCA, and to evaluate their physicochemical characteristics, stability, hemocompatibility, ROS-mediated photodynamic activity, effect on mitochondrial membrane potential (MMP), and anticancer efficacy against PC-3 cells. Molecular docking was performed to predict the interaction of CIP and LUT with 5α-reductase, AR, and TOP2A as biological targets involved in PCA progression

## 2. Results

### 2.1. LNP Formulations Design

LNP formulations were prepared using a constant lipid matrix of beeswax, coconut oil, and Pluronic F-68, while varying the incorporation of CIP, LUT, and CS coating. PLNPs served as the control, whereas CIPLNPs, LUTLNPs, CIPLUT-LNPs, and CCIPLUTLNPs were designed to evaluate the individual and combined effects of drug loading and CS coating on LNP performance. The beeswax–coconut oil lipid matrix provided a stable and biocompatible platform for drug delivery, with beeswax contributing a rigid hydrophobic core for improved CIP and LUT encapsulation and retention, and coconut oil enhancing lipid fluidity and LNP formation, thereby promoting efficient CIP and LUT encapsulation and LNP stability. The compositions of the developed LNP formulations are presented in Table 1.

### 2.2. The Physicochemical Characteristics and EE of LNPs

The physicochemical characterization of the prepared LNPs is presented in Table 2. All LNP formulations exhibited PS within the nanometer range (127.9–192.0 nm), indicating successful LNP fabrication. LUTLNPs displayed the lowest PS (127.9 ± 23.0 nm), whereas CCIPLUT-LNPs showed the largest size (192.0 ± 33.0 nm), reflecting the influence of dual drug loading and CS coating on particle dimensions. ZP analysis demonstrated that uncoated formulations possessed negative corona (−25.0 to −32.14 mV), while CS coated LNPs displayed positive surface charges (+30.30 to +33.40 mV), confirming successful surface modification. LNPs exhibited low PDI (0.262–0.356), indicating relatively homogeneous particle populations. Drug encapsulation efficiencies ranged from 74.65% to 84.56%, with LUTLNPs achieving the highest encapsulation efficiency, demonstrating efficient drug incorporation into the lipid matrix.

### 2.3. The In Vitro Release Study

**Figure 1:**
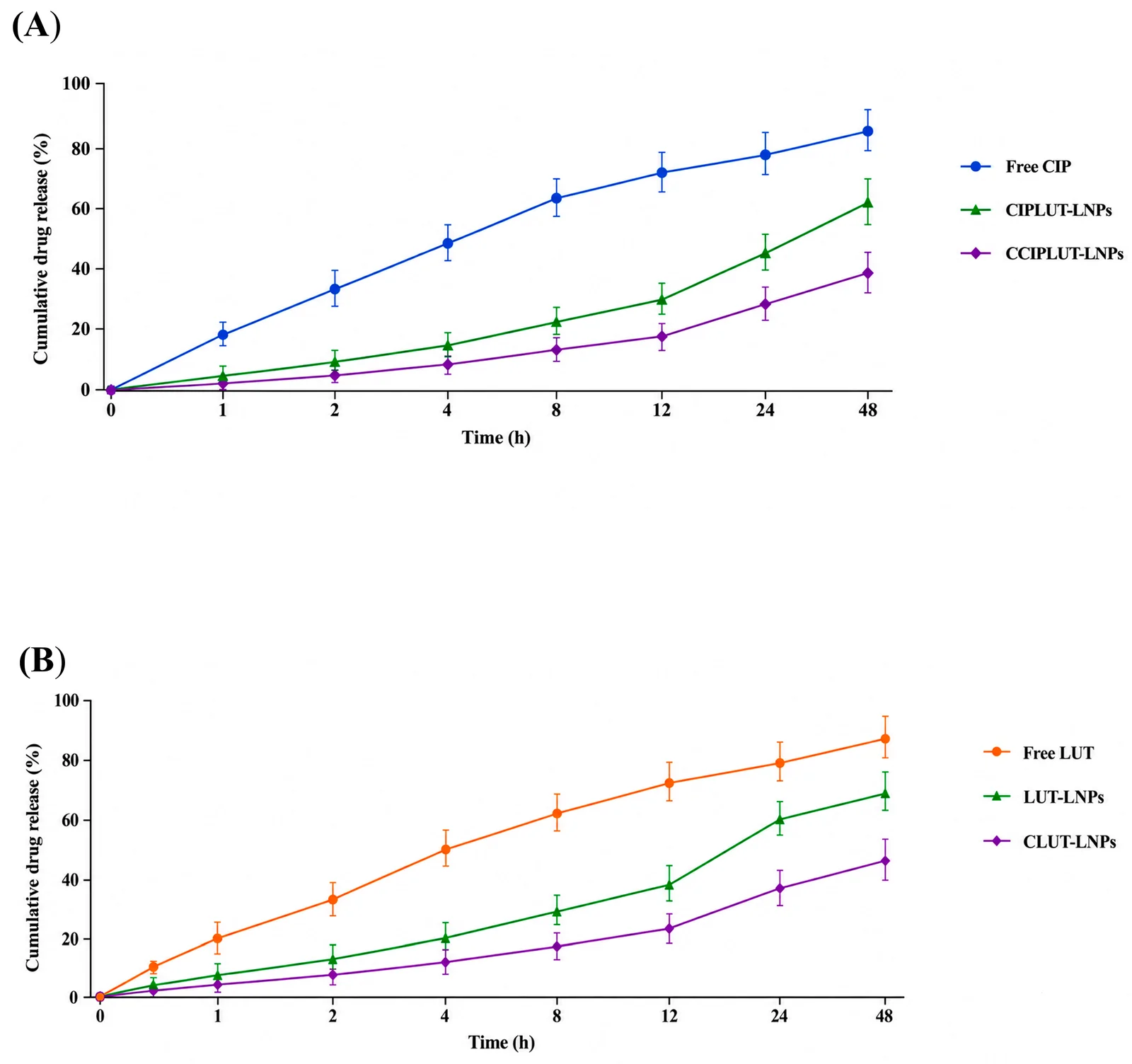
In vitro cumulative release profiles of CIP and LUT from the free drugs and corresponding LNP formulations over 48 h. (**A**): Release profiles of CIP, (**B**): Release profiles of LUT. Data are presented as mean ± SD (*n* = 3).

Figure 1A reveals the cumulative release profile of CIP. Free CIP exhibited rapid drug release, reaching approximately after 48 h, whereas CIPLUTLNPs showed a sustained release profile. CCIPLUTLNPs showed the slowest release, indicating that the CS coating effectively prolonged CIP release by providing an additional diffusion barrier. Figure 1B indicate the cumulative release profile of LUT. Free LUT was released rapidly, reaching approximately after 48 h. In contrast, both LUT-loaded LNP formulations exhibited controlled release, with CCIPLUTLNPs releasing significantly less LUT than CIPLU-LNPs throughout the study. These findings prove that CS coating effectively enhanced sustained release of LUT and minimized premature drug leakage.

### 2.4. Visual Stability of LNPs

The visual stability assessment following 3 months of LNP storage is summarized in Table 3. LNPs retained their natural color and homogeneous appearance throughout storage at 4 °C under light-protected conditions, with no evidence of irreversible phase separation or particle aggregation. Under ambient storage (25 ± 2 °C), only slight reversible phase separation and aggregation were observed, which disappeared after gentle shaking. Drug-loaded formulations containing LUT revealed a characteristic yellowish-white appearance, whereas LNPs without LUT remained milky white. Together, the prepared LNPs demonstrated satisfactory physical stability over the 3-month storage period, with refrigerated storage providing superior preservation of LNPs integrity.

### 2.5. Physicochemical Stability of LNPs

Changes in the physicochemical properties of the LNP formulations after 3 months of storage are presented in Table 4. Refrigerated storage at 4 °C resulted in only minimal increases in PS (1.4–2.2%), ZP (1.7–2.4%), and PDI (1.6–2.7%), together with negligible reductions in encapsulation efficiency (1.5–1.8%), indicating excellent physicochemical stability. In contrast, LNPs stored at ambient temperature presented moderately changes in PS (4.4–6.7%), ZP (5.7–7.6%), and PDI (6.2–9.2%), accompanied by modest reductions in EE (3.1–3.6%). Despite these changelings, the stability remained within acceptable stability limits. Among the tested formulations, CIPLUTLNPs and CCIPLUTLNPs demonstrated excellent stability during refrigerated storage, supporting their suitability for long-term storage under controlled conditions.

Stability Index (%) was calculated as 100 − average absolute percentage change in the evaluated physicochemical parameters (particle size, zeta potential, polydispersity index, and entrapment efficiency, where applicable). Higher values indicate better physicochemical stability after storage.

### 2.6. The Biocompatibility of the Developed LNPs

**Figure 2:**
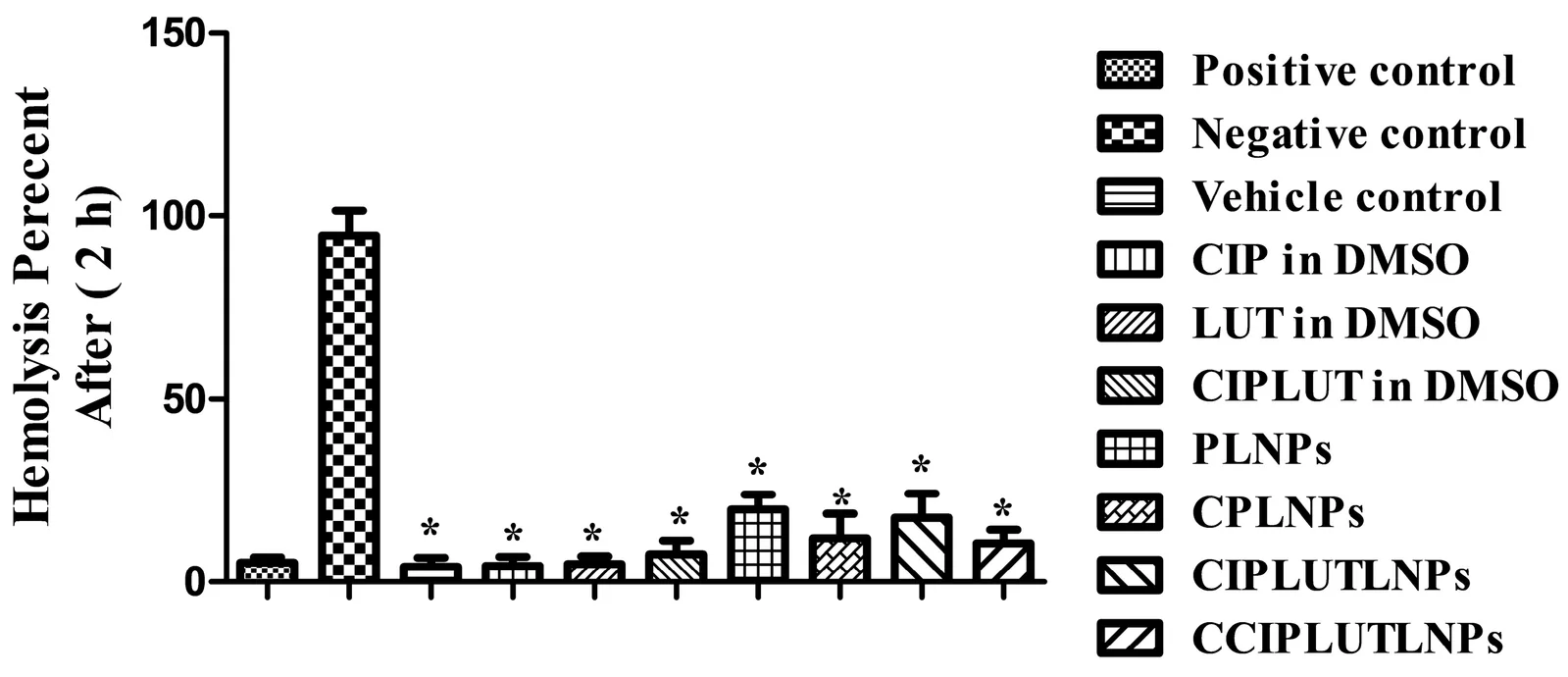
Quantitative analysis of the hemocompatibility of DMSO, free drugs, PLNPs, CIPLNPs, LUTLNPs, CIPLUTLNPs, and CCIPLUTLNPs relative to the positive and negative controls. Data are presented as the mean ± SD (*n* = 3). *, indicates a statistically significant reduction in hemolysis at *p* < 0.05, compared with the negative control, which induced complete hemolysis.

Quantitative analysis demonstrated that all LNPs produced significantly lower hemolysis than the negative control, indicating excellent blood compatibility. CS-coated LNPs maintained favorable hemocompatibility, representing that surface modification did not adversely affect erythrocyte integrity. See Figure 2.

### 2.7. Effect of LNPs on PC-3 Cells’ Viability

**Figure 3:**
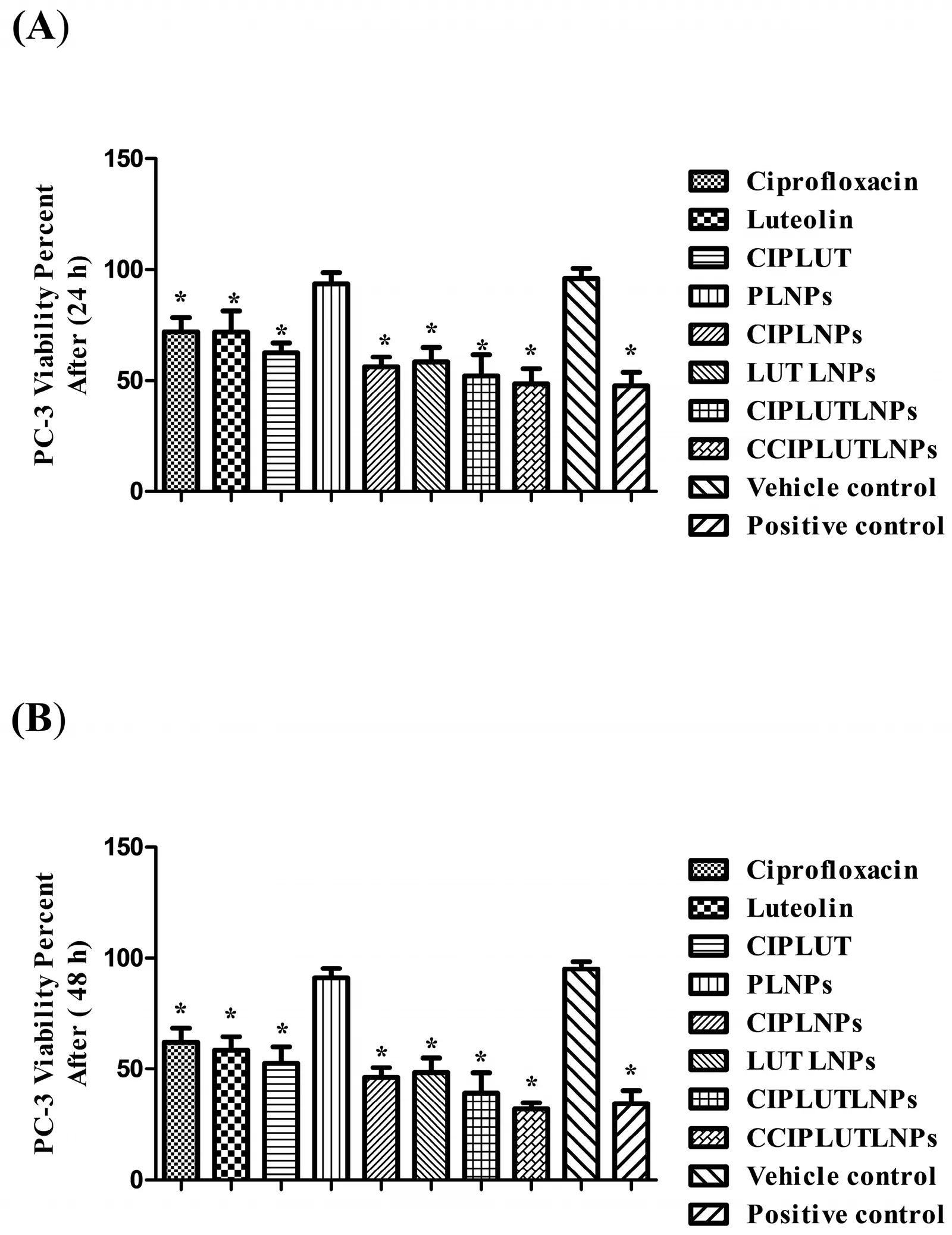
Effect of DMSO, free drugs, PLNPs, CIPLNPs, LUTLNPs, CIPLUTLNPs, and CCIPLUTLNPs on the viability of PC-3 cells following (**A**) 24 h and (**B**) 48 h of treatment. Data are presented as the mean ± SD (*n* = 3). *, indicates a statistically significant decrease in cell viability compared with the vehicle control group at *p* < 0.05.

Figure 3 displays the cytotoxic effects of free CIP, free LUT, their combination, PLNPs, drug-loaded LNPs, and CS-coated LNPs on PC-3 PCA cells after 24 h (Figure 3A) and 48 h (Figure 3B). After 24 h of treatment (Figure 3A), the vehicle control maintained approximately 100% cell viability, whereas the positive control noticeably reduced cell viability to about 45–50%. Blank PLNPs exhibited minimal cytotoxicity, with cell viability remaining close to 90%, confirming the biocompatibility of the LNPs. Free CIP and LUT moderately reduced PC-3 viability, while their combination (CIP–LUT) produced greater cytotoxicity than either drug alone, suggesting additive anticancer activity. Encapsulation of the drugs within LNPs significantly enhanced cytotoxicity compared with the corresponding free drugs. Among the LNPs, CIPLUTLNPs produced greater growth inhibition than the single drug-loaded LNPs, demonstrating the advantage of simultaneous drug co-delivery.

After 48 h of treatment (Figure 3B), all LNPs exhibited a further decrease in cell viability, indicating a clear time-dependent enhancement of cytotoxicity. PLNPs remained essentially non-toxic, whereas free CIP, free LUT, and their combination displayed moderate antiproliferative effects. The CIPLUTLNPs remained the most potent formulation, producing the lowest cell viability among all treatment groups and exhibiting anticancer activity nearly equivalent to the positive control.

### 2.8. ROS Generation Under Dark and Sunlight

**Figure 4:**
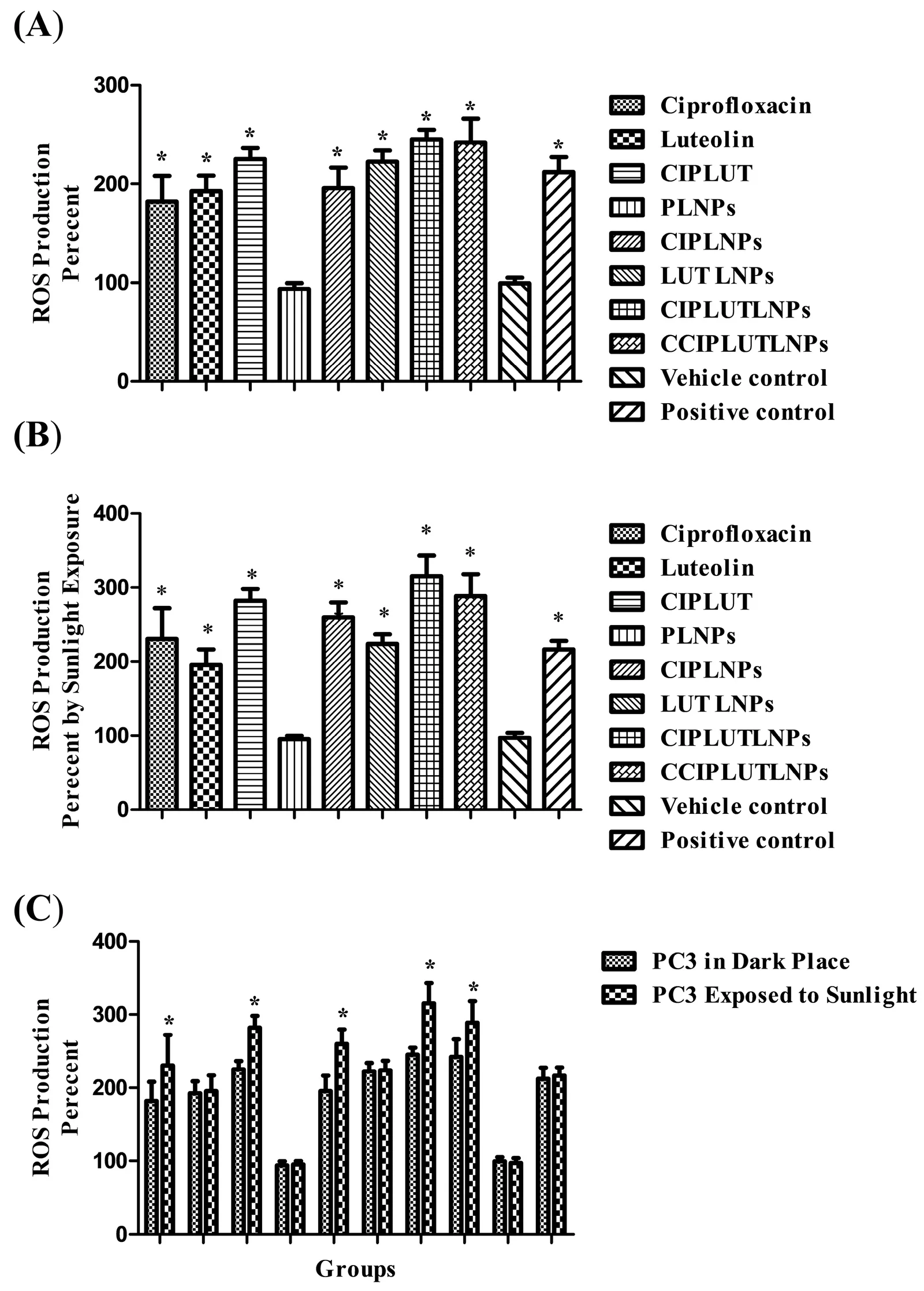
Effect of DMSO, free drugs, PLNPs, CIPLNPs, LUTLNPs, CIPLUTLNPs, and CCIPLUTLNPs on intracellular ROS generation in PC-3 cells. (**A**) Intracellular ROS production under dark conditions. (**B**) Intracellular ROS production following sunlight exposure. (**C**) Comparative analysis of intracellular ROS production under dark conditions and following sunlight exposure. Data are presented as the mean ± SD (*n* = 3). *, indicates a statistically significant increase in ROS levels compared with the vehicle control group at *p* < 0.05.

Figure 4 reveals that intracellular ROS production in PC-3 cells was influenced by both treatment type and light exposure. Under dark conditions (Figure 4A), free CIP and LUT significantly increased ROS levels compared with the vehicle control, with the free drug combination producing greater ROS than either drug alone. LNPs further enhanced ROS generation, although the increase remained moderate, indicating limited dark toxicity. CS coating did not markedly alter ROS production in the absence of light.

Following sunlight exposure (Figure 4B), ROS generation increased substantially, particularly in CIP-containing formulations, confirming the photosensitizing nature of ciprofloxacin. Encapsulation into LNPs enhanced the photodynamic activity of CIP, while co-loading with LUT further amplified ROS production. Both dual-loaded formulations (CIPLUTLNPs and CCIPLUTLNPs) produced the highest ROS levels, exceeding those of free drugs, plain LNPs, and the positive control.

Overall (Figure 4C), sunlight markedly potentiated CIP-mediated ROS generation, whereas LUT-containing LNP formulations alone produced only modest increases. The enhanced ROS production observed with the dual-loaded LNP formulations suggests synergistic effects of CIP, LUT, and LNP delivery, which may contribute to their superior anticancer activity through oxidative stress-mediated mechanisms, including apoptosis and ferroptosis.

### 2.9. Effect of LNPs on PC-3 Cells MMP

**Figure 5:**
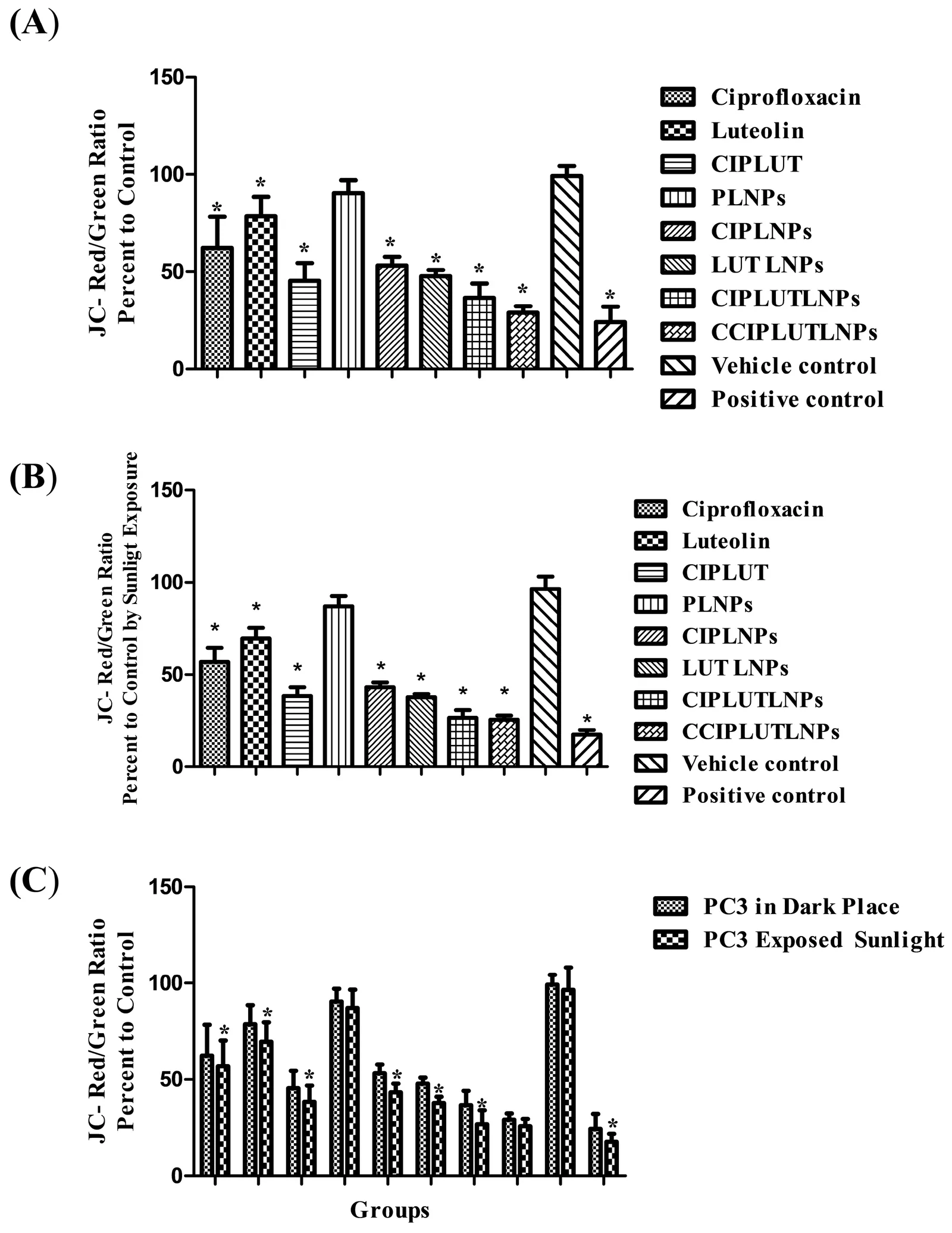
Effect of DMSO, free drugs, PLNPs, CIPLNPs, LUTLNPs, CIPLUTLNPs, and CCIPLUTLNPs on mitochondrial membrane potential (MMP) in PC-3 cells. (**A**) Quantitative analysis of MMP following treatment with the different formulations in dark condition. (**B**) Quantitative analysis of MMP following treatment with the different formulations following sunlight exposure. (**C**) comparative of MMP following treatment with the different formulations under dark conditions and following sunlight exposure. Data are presented as the mean ± SD (*n* = 3). *, indicates a statistically significant decrease in MMP compared with the vehicle control group at *p* < 0.05.

Figure 5A indicates MMP in dark conditions. In the lack of sunlight exposure, with free CIP, free LUT, CIPLUT, CIPLNPs, LUTLNPs, CIPLUTLNPs, and CCIPLUTLNPs significantly reduced the JC-1 red/green fluorescence ratio compared with the vehicle control, representing mitochondrial membrane depolarization. The highest decrease in MMP was noticed with the CCIPLUTLNPs, followed by CIPLUTLNPs, whereas free drugs and single-drug-loaded LNPs produced moderate decreases. PLNPs displayed minimal effects and showed values similar to the vehicle control, approving the biocompatibility of the blank carrier. The positive control produced the most pronounced loss of MMP.

Figure 5B reveals MMP by Sunlight exposure. Following sunlight irradiation, all CIP-containing LNP formulations exhibited greater mitochondrial depolarization compared with their corresponding dark-treated groups. CCIPLUTLNPs remained the most effective formulation, showing the lowest JC-1 red/green ratio, followed by CIPLUTLNPs, showing superior disruption of mitochondrial integrity after photoactivation. Free drugs also significantly reduced MMP, although to a lesser extent than the LNP formulations. Blank PLNPs again showed minimal effects, while the positive control induced the greatest mitochondrial damage.

Figure 5C shows comparison between dark and sunlight conditions. Direct comparison of the two experimental conditions demonstrates that sunlight exposure consistently improved MMP loss in all CIP-containing treatment groups, as reflected by lower JC-1 red/green fluorescence ratios than those observed under dark conditions. The difference was specifically manifest for CIPLNPs, CIPLUTLNPs, and CCIPLUTLNPs, highlighting the contribution of CIP-mediated PDT activation to mitochondrial dysfunction. In contrast, vehicle control and blank PLNPs showed little difference between dark and irradiated conditions.

In the sum, the CCIPLUTLNPs displayed the strongest ability to disrupt mitochondrial membrane potential, supporting its enhanced PDT and anticancer efficacy against PC-3 cells.

### 2.10. The Proposed Mechanism the Anticancer Mechanisms of CCIPLUTLNPs

**Figure 6:**
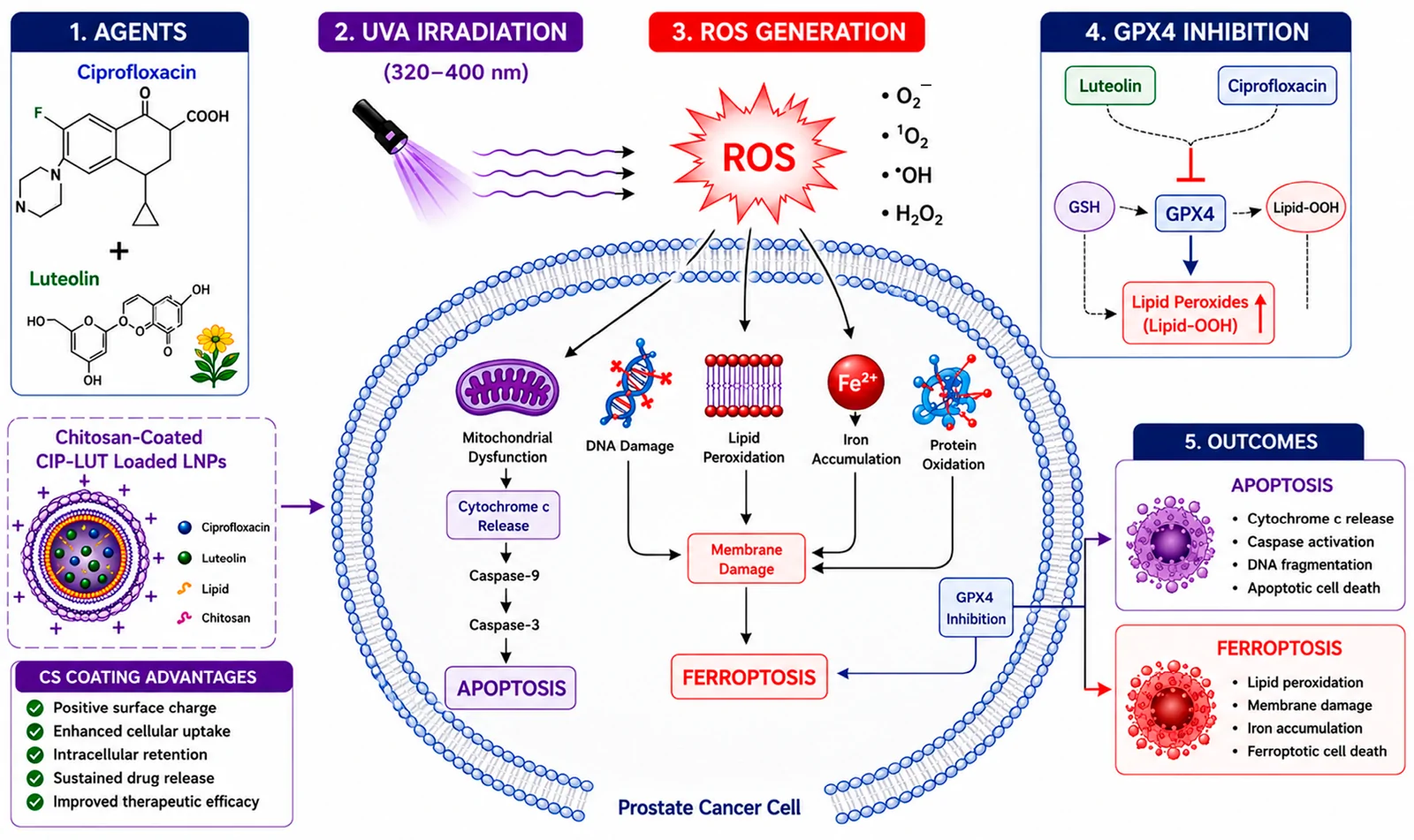
Schematic illustration of the proposed anticancer mechanism of CCIPLUTLNPs following UVA irradiation in prostate cancer cells. Following enhanced cellular uptake mediated by the positively charged chitosan coating, UVA irradiation photoactivates ciprofloxacin, while luteolin amplifies oxidative stress, leading to excessive ROS generation. The resulting oxidative damage induces mitochondrial dysfunction, lipid peroxidation, GPX4 inhibition, and activation of apoptotic and ferroptotic signaling pathways, ultimately promoting PCA cell death. In the schematic, arrows designate the proposed direction of molecular or cellular events, with black arrows representing mechanistic progression and colored arrows representing connections to the corresponding apoptotic or ferroptotic consequences. Red symbols and boxes denote oxidative stress, lipid peroxidation, membrane damage, and ferroptosis-related actions; purple elements represent mitochondrial dysfunction and apoptosis-related events; blue elements indicate GPX4-related processes; and green elements denote luteolin/chitosan-associated components. Molecular structures and icons represent the indicated therapeutic agents, cellular components, ROS species, and molecular pathway.

Figure 6 illustrate of the proposed mechanism underlying the anticancer activity of CCIPLUTLNPs against PCA cells. The positively charged CS coating promotes preferential interaction with the negatively charged, hypersialylated membrane of PCA cells, resulting in enhanced LNPs internalization and intracellular drug accumulation. Upon UVA irradiation (320–400 nm), photoactivated CIP generates excessive ROS, while LUT further amplifies oxidative stress. Elevated ROS induces mitochondrial dysfunction, DNA damage, protein oxidation, lipid peroxidation, membrane disruption, and iron overload, leading to activation of the intrinsic apoptotic pathway and ferroptosis. The simultaneous induction of these ROS-mediated cell death pathways contributes to enhanced PCA cell killing, highlighting the therapeutic potential of CCIPLUTLNPs as a targeted photodynamic nanoplatform for PCA therapy.

### 2.11. Molecular Docking Interaction Profiles of CIP and LUT

The molecular docking results are summarized in Table 5, demonstrating the interaction profiles of LUT and CIP with 5α-reductase, AR, and TOP2A. Both ligands established multiple hydrogen bonds and additional stabilizing interactions with key amino acid residues within the active sites of the investigated proteins. CIP displayed slightly stronger predicted binding affinities than LUT toward all three targets, with docking scores of −5.345, −7.860, and −7.692 kcal/mol for 5α-reductase, AR, and TOP2A, respectively. In comparison, LUT yielded docking scores of −5.106, −7.002, and −6.373 kcal/mol for the corresponding targets. The interaction analysis further revealed hydrogen bonding as the predominant binding mode, together with metal coordination and π-interactions where applicable, supporting the potential multi-target inhibitory activity of both compounds. Among the investigated proteins, the AR and TOP2A demonstrated the strongest predicted ligand binding, particularly for CIP, suggesting favorable target engagement relevant to PCA therapy.

**Figure 7:**
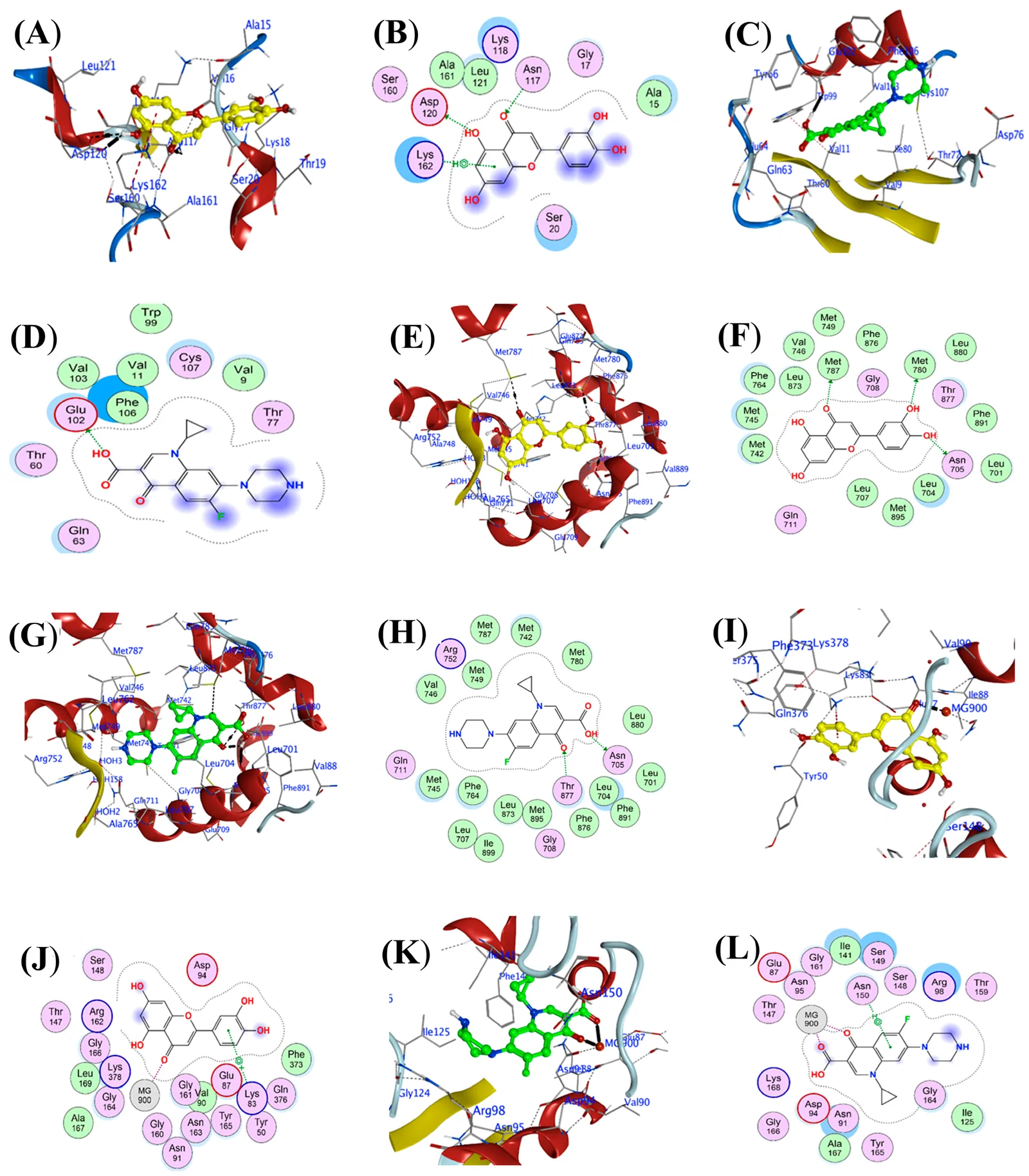
Molecular docking interaction maps of luteolin and ciprofloxacin with three prostate cancer-related molecular targets. Three-dimensional (3D) and two-dimensional (2D) binding interaction maps are shown for 5α-reductase with luteolin (**A**,**B**) and ciprofloxacin (**C**,**D**), androgen receptor (AR) with luteolin (**E**,**F**) and ciprofloxacin (**G**,**H**), and human DNA topoisomerase IIα with luteolin (**I**,**J**) and ciprofloxacin (**K**,**L**). For each target, the 3D and 2D interaction maps are presented in the first and second panels of each pair, respectively. In the 2D interaction maps, colored residue symbols indicate the physicochemical properties of the interacting amino acid residues, with purple, pink/red, blue, and light-green symbols representing polar, acidic, basic, and greasy/hydrophobic residues, respectively. Dashed arrows indicate hydrogen-bond interactions involving side-chain or backbone donor/acceptor groups, whereas dotted lines represent solvent contacts or metal/ion interactions. Green aromatic symbols indicate arene–arene, arene–H, and arene–cation interactions, as defined in the figure legend. Solid or shaded contours represent ligand or receptor exposure, respectively.

The molecular docking interactions of LUT and CIP with 5α-reductase are illustrated in Figure 7. Three-dimensional and two-dimensional interaction maps demonstrate that both ligands occupy the enzyme binding pocket and establish multiple stabilizing interactions with key amino acid residues. Hydrogen bonding, hydrophobic contacts, and van der Waals interactions contribute to ligand stabilization within the active site, supporting the potential inhibitory activity of both compounds toward 5α-reductase. The docking poses of LUT and CIP within the AR ligand-binding domain are presented in Figure 6. Both compounds exhibited favorable accommodation inside the receptor binding cavity and formed several non-covalent interactions with important amino acid residues. The interaction profiles indicate stable ligand binding and suggest that both compounds may interfere with androgen receptor signaling, which is closely associated with prostate cancer progression.

The docking interaction maps of LUT and CIP with human DNA TOP2A are shown in Figure 7. Both ligands displayed stable binding conformations within the enzyme active site through hydrogen bonds, hydrophobic interactions, and van der Waals contacts. These interaction patterns suggest favorable binding affinity toward DNA TOP2A, supporting its potential contribution as an additional molecular target underlying the anticancer activity of the investigated compounds

## 3. Discussion

This study successfully developed CS-coated lipid nanoparticles (CCIPLUTLNPs) for the co-delivery of CIP and LUT. The optimized formulation exhibited favorable physicochemical properties, excellent storage stability, high hemocompatibility, enhanced cytotoxicity against PC-3 prostate cancer cells, and favorable molecular interactions with multiple prostate cancer-associated therapeutic targets. Collectively, these findings identify CCIPLUTLNPs as a promising multifunctional nanoplatform for prostate cancer therapy, consistent with our previous work on LNPs-based targeted drug delivery systems for PCA [4].

The formulation strategy employed a constant lipid matrix consisting of beeswax and coconut oil while varying drug loading and CS coating to evaluate the contribution of each component to LNPs performance. All LNPs displayed nanosized PS with low PDI values, indicating successful fabrication and good formulation homogeneity. Nanoparticles within this size range facilitate cellular uptake via endocytosis and may improve tumor accumulation through the enhanced permeability and retention (EPR) effect [22,23]. CS coating resulted in a slight increase in PS together with a reversal of ZP from negative to positive, confirming successful surface modification. The positively charged CS layer enhances electrostatic interactions with negatively charged PCA cell membranes while improving colloidal stability by reducing nanoparticle aggregation [4,19,24]. Drug encapsulation efficiencies of approximately 75–85% indicate efficient incorporation of both drugs into the lipid matrix. The higher encapsulation efficiency of LUT is likely attributable to its hydrophobicity, whereas CIP was effectively retained through interactions with lipid and surfactant components. Efficient encapsulation minimizes premature drug leakage and supports sustained drug release, thereby improving therapeutic efficacy.

All of the prepared LNP formulations remained physically stable throughout three months of storage. Refrigerated storage produced only minimal changes in PS, PDI, ZP, and EE, whereas slightly greater but acceptable variations were observed under ambient conditions. These findings agree with previous reports identifying these parameters as key indicators of LNP stability [23]. Minor phase separation observed at room temperature was readily reversible upon gentle shaking, indicating that irreversible instability did not occur and supporting refrigerated storage as the preferred condition for long-term preservation. In addition, all LNPs exhibited excellent hemocompatibility, with negligible hemolysis compared with the positive control. Importantly, CS coating did not compromise blood compatibility despite introducing a positive surface charge, indicating that the degree of cationic modification remained biologically acceptable [4,19,24,25].

Biological evaluation demonstrated that drug-loaded LNPs significantly enhanced cytotoxicity against PC-3 cells in both concentration- and time-dependent manners, with maximal activity after 48 h. Dual drug-loaded formulations were more effective than either free drug alone, while CCIPLUTLNPs exhibited the greatest antiproliferative activity, comparable to the positive control. The enhanced efficacy is attributed to improved intracellular drug delivery, sustained drug release, and the positively charged CS coating, which promotes electrostatic interaction with negatively charged PC-3 cell membranes and enhances LNPs internalization. These findings are consistent with previous studies demonstrating improved anticancer efficacy following nanoencapsulation of CIP and other therapeutic agents [4,19,24,25]. The superior activity of CCIPLUTLNPs is also supported by the complementary mechanisms of the incorporated drugs. CIP exerts anticancer activity through inhibition of TOP2A, disruption of microtubule polymerization, suppression of angiogenesis, and modulation of multidrug resistance pathways [8,9]. Meanwhile, LUT regulates multiple signaling pathways, including PI3K/AKT/mTOR, MAPK, NF-κB, and AR signaling, while promoting apoptosis and ferroptosis [11,13]. Simultaneous delivery of both agents therefore enables multi-target therapeutic intervention, resulting in synergistic suppression of PCA cell proliferation.

Beyond serving as structural components, the lipid matrix may provide additional therapeutic benefits. Beeswax has demonstrated antiproliferative activity against PCA cells, whereas coconut oil-derived medium-chain fatty acids modulate oxidative stress, mitochondrial function, and lipid metabolism, processes closely associated with PCA development [26,27]. Consequently, the lipid matrix may complement the anticancer activities of CIP and LUT while supporting LNPs stability and drug encapsulation.

ROS generation represented one of the principal mechanisms underlying the observed anticancer activity. CCIPLUT-LNPs induced the greatest intracellular ROS accumulation, indicating enhanced oxidative stress compared with the corresponding free drugs. Although physiological ROS regulate normal cellular signaling, excessive ROS damages proteins, lipids, DNA, and mitochondria, ultimately triggering apoptosis. In addition to directly inducing oxidative stress, CIP generates ROS following UVA irradiation, whereas LUT exhibits concentration-dependent pro-oxidant activity in cancer cells [8,9,13]. Furthermore, LUT promotes ferroptosis through GPX4 inhibition, glutathione depletion, iron accumulation, and lipid peroxidation, thereby disrupting redox homeostasis and amplifying ROS-mediated cytotoxicity [11,13,28,29,30]. These complementary mechanisms likely account for the superior ROS generation and cytotoxicity observed with CCIPLUTLNPs.

The ROS findings further confirmed the photodynamic potential of CIP. Under dark conditions, all treatment groups generated relatively low ROS levels, indicating minimal oxidative stress and limited photoactivity. In contrast, sunlight irradiation markedly increased ROS production, particularly in CIP-containing formulations, confirming the intrinsic photosensitizing properties of CIP. Upon UVA exposure, excited-state CIP undergoes Type I and Type II photochemical reactions that generate superoxide radicals, hydroxyl radicals, and singlet oxygen, resulting in oxidative damage to cellular lipids, proteins, DNA, and mitochondria [31,32,33,34]. Among all LNP formulations, CCIPLUTLNPs produced the highest ROS levels after irradiation, likely owing to enhanced intracellular uptake, prolonged retention, and the pro-oxidant activity of LUT within cancer cells. The noticeable difference between dark and irradiated conditions confirms a predominantly light-triggered mechanism, supporting the potential of CCIPLUTLNPs as an effective photodynamic nanomedicine for prostate cancer.

Drug-loaded LNPs also significantly reduced MMP, indicating activation of the intrinsic apoptotic pathway. CCIPLUTLNPs induced the greatest mitochondrial depolarization, consistent with enhanced intracellular delivery mediated by the positively charged CS coating. Previous studies similarly demonstrate that both CIP and LUT promote mitochondrial dysfunction and apoptosis, whereas LUT additionally induces ferroptosis, collectively contributing to enhanced cancer cell death [9,13,35].

Molecular docking further supported the experimental findings by demonstrating favorable binding of both CIP and LUT to three therapeutically relevant proteins, namely 5α-reductase, AR, and TOP2A. These interactions suggest simultaneous inhibition of androgen signaling and DNA replication pathways, providing a mechanistic basis for the multitarget therapeutic effects observed experimentally. Collectively, the computational and biological findings indicate that the superior anticancer activity of CCIPLUTLNPs results from coordinated modulation of multiple molecular pathways rather than a single therapeutic target [36,37].

In sum, this study presents the first CS-coated CIPLUT lipid nanoplatform integrating drug repurposing, nanomedicine, and PDT for PCA. Nanoparticle encapsulation enhanced the photosensitizing activity of CIP, while co-delivery with LUT synergistically increased ROS generation, mitochondrial dysfunction, and PC3 cell death. Together with favorable physicochemical characteristics, excellent stability, high hemocompatibility, and multitarget molecular interactions, these findings establish CCIPLUTLNPs as a promising ROS-mediated therapeutic strategy for prostate cancer and warrant further evaluation using clinically relevant irradiation systems and in vivo models.

## 4. Materials and Methods

### 4.1. Materials

Luteolin (LUT) was purchased from Sigma-Aldrich (St. Louis, MO, USA). Ciprofloxacin (CIP) was kindly given as a gift sample by Spimaco Aldawaeia (Riyadh, Saudi Arabia). CS low molecular weight, Tween, and Pluronic F-68 were obtained from Sigma-Aldrich (St. Louis, MO, USA). All of the remaining chemicals are of high purity.

### 4.2. Development of CIPLUTNLCs

Drug-free plain LNPs (PLNPs), LUT-loaded LNPs (LUTLNPs), and CIP-loaded LNPs (CIPLNPs) were prepared using the ultrasonic melt-emulsification method. The lipid phase consisted of beeswax as the solid lipid and coconut oil as the liquid lipid, whereas the aqueous phase comprised Pluronic^®^ F-68 (St. Louis, MO, USA) dissolved in distilled water. The compositions of the formulations, including the amounts of beeswax, coconut oil, CIP, and LUT, are presented in Table 1. Briefly, the lipid components and the appropriate amount of drug(s) were weighed and mixed to prepare the lipid phase. Separately, Pluronic^®^ F-68 was dissolved in distilled water to prepare the aqueous phase. The aqueous phase was initially maintained at 4 °C to ensure complete dissolution before both phases were heated separately to 80 °C. The heated aqueous phase was then added to the molten lipid phase under continuous stirring to form a hot pre-emulsion. The resulting pre-emulsion was subjected to probe sonication (ATP-150, Mumbai, India) at 80% amplitude for six cycles of 40 s each, with 5 s intervals between cycles, to obtain the lipid nanoparticles. Formation of the nanoparticles was indicated by the development of a stable off-white dispersion. The prepared formulations were characterized for particle size (PS), polydispersity index (PDI), and zeta potential (ZP), and subsequently stored at 4 °C until further analysis [4,19,38].

### 4.3. Cationic Engineering of CIPLUTLNPs

CS-coated LNPs (CPLNPs) and CS-coated CIP–LUT-loaded LNPs (CCIPLUTLNPs) were prepared by electrostatic deposition of CS onto the surface of the corresponding uncoated formulations. Briefly, equal volumes of PLNPs or CIPLUTLNPs were mixed with a CS solution prepared in 0.5% (*v*/*v*) acetic acid. The CS solution was added dropwise to the nanoparticle dispersion under continuous magnetic stirring (C-MAG HS 10, IKA, Staufen, Germany) for 20 min to facilitate uniform coating. The resulting CS-coated lipid nanoparticles were then stirred continuously for an additional 2 h to ensure complete adsorption of the polymer onto the nanoparticle surface. Both uncoated and CS-coated LNP formulations were stored at 4 °C until further characterization and subsequent use [4,19,24,38]. 

### 4.4. LNPs PS, PDI, and ZP Measurement

The particle size (PS), polydispersity index (PDI), and zeta potential (ZP) of all LNP formulations were determined using a Zetasizer Nano ZS (Malvern Instruments, Worcestershire, UK). Prior to analysis, each formulation was diluted 1:1000 with phosphate-buffered saline (PBS) to obtain an appropriate measurement concentration. All measurements were performed at 25 °C under standardized conditions. Each sample was analyzed in triplicate, and the results are presented as the mean ± standard deviation (SD) to ensure the accuracy and reproducibility of the measurements [4,19,24].

### 4.5. CIP and LUT Entrapment Efficiency

The entrapment efficiency (EE) of CIP and LUT into LNPs was evaluated using the indirect method of centrifugation techniques. Each CIP and LUT in LNPs (2 mL) was transferred to the tube and centrifuged at 10,000 rpm for 1 h using a cooling centrifuge (Centurion Scientific Limited, West Sussex, UK). After completion, the supernatant was collected, filtered, and further diluted with methanol to measure CIP and LUT at 278 and 343 nm using UV spectroscopy (Shimadzu 1701, Kyoto, Japan). The absorbance was used to calculate the amount of drug encapsulated in the LNPs using the CIP and LUT standard curve.

$$Entrapment\;effeciency\;(\%)=\frac{Total\;drug-Free\;drug}{Total\;drug}\times 100$$

### 4.6. The In Vitro Release Study

The in vitro release profiles of CIP and LUT from LNPs and CS-coated LNPs (were evaluated using the dialysis bag diffusion method [38]. Briefly, each formulation was placed into a pre-swollen dialysis membrane (molecular weight cut-off (MWCO): 8 kDa) and immersed in phosphate-buffered saline (PBS, pH 7.4) containing 0.5% Tween 80. The release study was conducted in a water-bath shaker (JULABO GmbH, Seelbach, Germany) maintained at 37 ± 0.5 °C with continuous shaking at 100 rpm. At predetermined time intervals, 3 mL of the release medium was withdrawn and immediately replaced with an equal volume of fresh prewarmed medium to maintain sink conditions. The collected samples were filtered through a 0.22 μm membrane filter, and the absorbance of CIP and LUT was measured using a UV–Vis spectrophotometer. The cumulative amount of drug released was calculated from the corresponding calibration curves. All experiments were performed in triplicate, and the results are presented as mean ± standard deviation (SD).

### 4.7. Stability Assessment of LNPs

The stability of blank LNPs and CIPLUTLNPs was evaluated under different storage conditions (4 °C and 25 °C, with and without light protection) for up to 3 months. At weekly intervals, formulations were visually inspected for aggregation, sedimentation, color changes, and phase separation. Physicochemical stability was assessed by measuring PS, PDI, and ZP using dynamic light scattering to monitor colloidal stability and structural integrity.

### 4.8. LNPs Biocompatibility Screening

The hemocompatibility of free drug, PLNPs, LUTLNPs, CIPLNPs, CPLNPs, CIPLUTLNPs, and CCIPLUTLNPs was evaluated using an in vitro hemolysis assay. Whole blood (2 mL) was collected from male rats by cardiac puncture immediately following euthanasia. All animal procedures were approved by the Research Ethics Committee (REC) of King Saud University, Riyadh, Saudi Arabia (Ref. No. KSU-SE-26-23), and were conducted in accordance with institutional and international ethical guidelines. Euthanasia was performed by intraperitoneal administration of ketamine (80 mg/kg) and xylazine (10 mg/kg).

The collected blood was transferred into heparinized tubes and centrifuged at 3000 rpm for 5 min. Plasma and the buffy coat were carefully removed, and the erythrocytes were washed three times with cold isotonic Ringer’s solution, with centrifugation at 3000 rpm for 5 min after each wash. The purified erythrocytes were then resuspended in Ringer’s solution to obtain a hematocrit of 10%.

The erythrocyte suspension was divided into the following experimental groups: (1) Positive control, erythrocytes incubated with phosphate-buffered saline (PBS); no hemolytic effect on erythrocytes. (2) Negative control, erythrocytes treated with 1% Tween in distilled water to induce complete hemolysis. (3) Vehicle control, erythrocytes exposed to 1% DMSO. (4) Free CIP dissolved in 1% DMSO. (5) Free LUT dissolved in 1% DMSO; and (6–10) erythrocytes incubated with PLNPs, LUTLNPs, CPLNPs, CIPLUTLNPs, and CCIPLUTLNPs, respectively.

Following gentle mixing, all samples were incubated at 37 °C for 2 h and subsequently centrifuged at 3000 rpm for 5 min using a Centrifuge EBA 20 (Hettich, Tuttlingen, Germany). The absorbance of the supernatant was measured at 570 nm using a UV–Vis spectrophotometer (Genesys 10S UV–Vis, Thermo Scientific, Waltham, MA, USA). The percentage of hemolysis was calculated using the following equation [4,19,24].

$$Hemolysis\;ratio\;(\%)=\frac{Absorbance\;sample-absorbance\;negative\;control}{absorbance\;positive\;control-absorbance\;negative\;control}\times 100$$

### 4.9. Cell Culture and Cell Viability Assay

The human prostate cancer cell line (PC-3) was used as an in vitro model of prostate cancer (PCA). PC3 cells were acquired from the American Type Cell Culture (ATCC, Manassas, VA, USA). Cells were cultured in Dulbecco’s Modified Eagle Medium (DMEM) supplemented with 10% fetal bovine serum (FBS) and 1% penicillin–streptomycin and maintained at 37 °C in a humidified incubator containing 5% CO_2_ and 95% air. For the cell viability assay, PC-3 cells were seeded into 96-well plates at a density of 5 × 10^3^ cells/well and allowed to adhere for 24 h. Following incubation, the cells were treated with free CIP, free LUT, their combination (CIP + LUT), PLNPs, LUTLNPs, CIPLNPs, CIPLUTLNPs, and CCIPLUTLNPs. Cells treated with 1% DMSO served as the vehicle control, whereas those treated with gefitinib (dissolved in 1% DMSO) served as the positive anticancer control. After treatment for 24 and 48 h, cell viability was determined using the methyl thiazolyl tetrazolium (MTT) assay according to the manufacturer’s instructions. All treatments were performed in triplicate, and cell viability was expressed as a percentage relative to the vehicle control.

$$Viability\left(\%\right)=\frac{optical\;density\;of\;treated\;group}{optical\;density\;of\;untreated\;group}\times 100$$

### 4.10. ROS Generation and Photodynamic Irradiation

Intracellular reactive ROS generation was measured using the H_2_DCFDA Cellular ROS Assay Kit (MedChemExpress, Monmouth Junction, NJ, USA; Cat. No. HY-D0940), which employs the cell-permeable fluorescent probe 2′,7′-dichlorodihydrofluorescein diacetate (H_2_DCFDA) to detect intracellular ROS. PC-3 cells were cultured in Dulbecco’s Modified Eagle Medium (DMEM) supplemented with 10% fetal bovine serum (FBS) and 1% penicillin–streptomycin at 37 °C in a humidified incubator containing 5% CO_2_. Upon reaching 70–80% confluence, the cells were detached using 0.25% trypsin–0.02% EDTA (Gibco, Thermo Fisher Scientific, Waltham, MA, USA), neutralized with complete culture medium, collected by centrifugation at 1200 rpm for 5 min, and resuspended in prewarmed complete medium. Cell viability and density were determined using a Countess II Automated Cell Counter (Thermo Fisher Scientific, Waltham, MA, USA), before seeding.

PC-3 cells were seeded into 96-well plates at a density of 3 × 10^4^ cells/well in 100 μL of complete medium and incubated for 24 h to allow cell attachment. The cells were then treated with free CIP, free LUT, their combination (CIP + LUT), PLNPs, LUTLNPs, CIPLNPs, CIPLUTLNPs, or CCIPLUTLNPs. Cells treated with 1% DMSO served as the vehicle control, whereas gefitinib (prepared in 1% DMSO) was used as the positive anticancer control. Following 1 h of treatment, the culture medium was removed, the cells were washed twice with PBS, and fresh serum-free medium was added.

For photodynamic activation, one set of plates was maintained under standard culture conditions in the dark as the non-irradiated control, while a second set was exposed to direct midday sunlight on clear days between 12:00 PM and 2:00 PM for 1 h at an ambient temperature of 25 ± 2 °C, according to previously reported photoactivation protocols [32]. During irradiation, the plates were placed on a platform surrounded by ice packs to minimize thermal effects and ensure that the observed responses resulted primarily from sunlight-induced photoactivation of CIP.

Immediately after the dark incubation or sunlight exposure, intracellular ROS levels were determined according to the manufacturer’s instructions using the H_2_DCFDA assay. Oxidation of H_2_DCFDA to fluorescent dichlorofluorescein (DCF) was quantified using a microplate reader (BMG LABTECH Ltd., Ortenberg, Germany) at excitation and emission wavelengths of 485 and 529 nm, respectively. Fluorescence intensity was used as an indicator of intracellular ROS production, and ROS levels in sunlight-irradiated cells were directly compared with those of matched non-irradiated control cells maintained under identical culture conditions in the dark.

### 4.11. MMP Assay

MMP was evaluated using MMP Assay Kit (MedChemExpress, Monmouth Junction, NJ, USA; Cat. No. HY-K0601) according to the manufacturer’s instructions. The assay is based on the membrane-permeable fluorescent probe JC-1, which accumulates in polarized mitochondria as red-fluorescent aggregates, whereas in depolarized mitochondria it remains in its monomeric form, emitting green fluorescence.

PC-3 cells were seeded into 96-well plates at a density of 2 × 10^4^ cells/well and incubated for 24 h at 37 °C in a humidified atmosphere containing 5% CO_2_ to allow cell attachment. The cells were treated with free CIP, free LUT, their combination (CIP + LUT), PLNPs, LUTLNPs, CIPLNPs, CIPLUTLNPs, and CCIPLUTLNPs. Cells treated with 1% DMSO served as the vehicle control, whereas those treated with gefitinib (dissolved in 1% DMSO) served as the positive anticancer control. Following treatment, the cells were incubated with 2 μM JC-1 reagent for 15 min at 37 °C. Fluorescence was measured using a microplate reader at excitation/emission wavelengths of 530/590 nm for JC-1 aggregates (red fluorescence) and 485/528 nm for JC-1 monomers (green fluorescence). For PDT irradiation the microplate was processed as ROS experiment. The ratio of red-to-green fluorescence intensity was calculated for each treatment group as an indicator of MPP, with a reduction in the ratio reflecting mitochondrial depolarization.

### 4.12. Molecular Docking Studies

Molecular docking studies were carried out using MOE 2024.06. The 3D structures of CIP and LUT were built and prepared using MOE’s standard ligand preparation protocol. X-ray crystal structures of biologically relevant target proteins were retrieved from the Protein Data Bank and used as receptors. The molecular docking analysis focused on three protein targets with well-established biological and therapeutic importance: 5α-reductase, AR, and TOP2A. The crystal structure of human 5α-reductase (PDB ID: 6KX3, resolution 1.98 Å) was selected due to its central role in androgen metabolism and relevance in androgen-related disorders. The AR, a key regulator of androgen signaling and a critical target in PCA, was represented by its high-resolution X-ray structure (PDB ID: 1T7F, 1.60 Å), ensuring accurate modeling of ligand–receptor interactions. Additionally, TOP2A, an essential enzyme involved in DNA replication and cell division and a prominent anticancer drug target, was modeled using the crystal structure 1ZXN with a resolution of 2.51 Å. These ligand-bound, high-quality structures were chosen to provide reliable and biologically relevant binding sites for molecular docking studies.

Docking sites were defined based on the positions of co-crystallized ligands, ensuring biologically validated binding pockets. Each ligand was docked using the Triangle Matcher algorithm, generating 1000 poses scored by the London ΔG function, followed by rescoring the top 100 poses with the GBVI/WSA ΔG function. Receptor flexibility was considered through the Induced Fit refinement protocol. The docking protocol was validated by redocking co-crystallized ligands into their respective binding sites. Accuracy was confirmed by RMSD analysis, with values below 2.0 Å demonstrating reliable reproduction of experimental binding modes.

### 4.13. Statistical Analysis

Data analysis was achieved by GraphPad software, version 5 (GraphPad, ISI Software Inc., La Jolla, CA, USA). The results were compared using a one-way analysis of variance. Data were expressed as mean ± SD, and *p*-values < 0.05 were used as criteria for significance.

## 5. Conclusions

The current work successfully developed CCIPLUTLNPs as a multifunctional nanoplatform for PCA therapy. The LNP formulations displayed desirable physicochemical characteristics, including nanoscale PS, narrow PDI, high EE, and enhanced physicochemical stability, demonstrating the beneficial effect of CS coating on LNPs performance. Compared with conventional formulations, the CS-coated system provided improved colloidal stability and is expected to enhance cellular interaction through the mucoadhesive and positively charged properties of CS. Biologically, CCIPLUTLNPs significantly enhanced anticancer activity against PC-3 cells, indicating superior cytotoxicity compared with the free drugs and non-coated formulations. The LNP formulations noticeably increased intracellular ROS generation, induced marked mitochondrial membrane depolarization, and substantially improved PDT efficacy under sunlight irradiation, indicating enhanced mechanism of ROS-mediated tumor cell death. These findings suggest that combining drug repurposing with nanotechnology and PDT may represent an effective strategy for improving PCA treatment while potentially reducing the required therapeutic dose and minimizing off-target effects. Furthermore, molecular docking analyses established favorable interactions of CIP and LUT with multiple PCA-related targets, including 5α-reductase, AR, and human TOP2A, supporting the proposed multitarget therapeutic mechanism of the developed formulation. These computational findings complement the experimental results and provide mechanistic insight into the enhanced anticancer activity observed in vitro.

Overall, the developed CCIPLUTLNPs represent a promising multifunctional nanoplatform that integrates targeted drug delivery, PDT, and multitarget molecular interactions to advance PCA treatment. Although the present findings are encouraging, further studies involving detailed mechanistic investigations, pharmacokinetic and biodistribution analyses, in vivo efficacy and safety evaluations, and ultimately clinical validation are warranted to confirm the translational potential of this therapeutic strategy. This work provides a strong foundation for the future development of CS-coated LNPs as advanced nanomedicines for PCA management.

### Study Limitations

Although the present work represents a promising result and providing in vitro evidence for the potential of CCIPLUTLNPs as a multifunctional PDT nanoplatform for PCA management, several limitations should be acknowledged. The morphology of the LNPs was not confirmed by SEM/TEM, and the biological evaluation was limited to a single PCA cell line (PC-3) without the inclusion of a normal prostate epithelial cell line. The PDT experiments employed natural sunlight rather than a standardized light source; therefore, future studies should utilize standardized light sources with controlled wavelength, irradiance, and fluence to improve reproducibility. Moreover, the proposed involvement of ferroptosis was not directly validated using specific biomarkers such as GPX4, lipid peroxidation, or intracellular iron levels. In addition, the enhanced cellular uptake of CS-coated LNPs was inferred from their positive surface charge and biological activity but was not directly confirmed using cellular uptake studies, such as fluorescence microscopy or flow cytometry. Furthermore, the molecular docking results require biochemical validation. Ultimately, further in vivo evaluation is warranted to establish the therapeutic efficacy, safety, mechanism of action, and translational potential of the developed CS coated CIPLUT-loaded LNPs.

## Figures and Tables

**Table 1:** Components of the prepared lipid nanoparticle formulations.

Ingredients/LNPs	PLNPs	CPLNPs	CIPLNPs	LUTLNPs	CIPLUTLNPs
Beeswax (mg)	350	350	350	350	350
Coconut oil (mg)	150	150	150	150	150
Pluronic F-68 (mg)	350	350	350	350	350
Ciprofloxacin	-	-	50	-	50
Luteolin (µg)	-	-	-	50	50
Distilled water (mL)	10	10	10	10	10
CS solution (mL)	-	+	-	-	-

**Table 2:** The particle size (PS), zeta potential (ZP), and polydispersity index (PDI), of PLNPs, CPLNPs, CIPLNPs LUTLNPs, and CIPLUT-LNPs.

	PLNPs	CPLNPs	CIPLNPs	LUTLNPs	CIPLUTLNPs	CCIPLUTLNPs
PS (nm)	166.0 ± (35.0)	178.0 ± (24.0)	185.0 ± (21.0)	127.9 ± (23.0)	182.0 ± (33.0)	192.0 ± (33.0)
ZP (mV)	−30.30 ± (7.00)	33.40 ± (6.00)	−32.14 ± (5.00)	−25.0 ± (−4.00)	−31.30 ± (5.00)	30.30 ± (5.00)
PDI	0.288 ± (0.033)	0.276 ± (0.034)	0.314 ± (0.044)	0.262 ± (0.045)	0.314 ± (0.045)	0.356 ± (0.035)
EE (%)	-	-	74.82 ± 7.45	84.56 ± 9.27	74.65 ± 6.89	79.65 ± 7.89

**Table 3:** Visual stability of lipid nanoparticle formulations after 3 months of storage under different storage conditions.

Formulation	Storage Condition	Color	Phase Separation	Aggregation	Overall Stability
PLNPs	4 °C, light-protected	Milky white	None	None	Stable
	25 ± 2 °C, ambient light	Milky white	Slight reversible	Slight reversible	Acceptable stability
CPLNPs	4 °C, light-protected	Milky white	None	None	Stable
	25 ± 2 °C, ambient light	Milky white	Slight reversible	Slight reversible	Acceptable stability
CIPLNPs	4 °C, light-protected	Milky white	None	None	Stable
	25 ± 2 °C, ambient light	Milky white	Minimal reversible	Slight reversible	Acceptable stability
LUTLNPs	4 °C, light-protected	Yellowish-white	None	None	Stable
	25 ± 2 °C, ambient light	Yellowish-white	Minimal reversible	Slight reversible	Acceptable stability
CIPLUTLNPs	4 °C, light-protected	Yellowish-white	None	None	Stable
	25 ± 2 °C, ambient light	Yellowish-white	Minimal reversible	Slight reversible	Acceptable stability
CCIPLUTLNPs	4 °C, light-protected	Yellowish-white	None	None	Stable
	25 ± 2 °C, ambient light	Yellowish-white	Minimal reversible	Slight reversible	Acceptable stability

During three-month storage period. All formulations maintained their original color and homogeneous appearance with no irreversible phase separation or aggregation. Minor reversible phase separation and aggregation observed at 25 ± 2 °C were readily dispersed by gentle shaking, indicating acceptable physical stability.

**Table 4:** Percentage changes in physicochemical properties and stability index (%) of lipid nanoparticle formulations after 3 months of storage under different storage conditions.

Formulation	Storage Condition	ΔPS (%)	ΔZP (%)	ΔPDI (%)	ΔEE (%)	Stability Index (%)
PLNPs	4 °C, light-protected	+1.9	2.0	+2.1	—	98.0
	25 ± 2 °C, ambient light	+4.7	6.3	+7.3	—	93.9
CPLNPs	4 °C, light-protected	+2.0	1.8	+1.8	—	98.1
	25 ± 2 °C, ambient light	+4.4	5.7	+6.9	—	94.3
CIPLNPs	4 °C, light-protected	+1.8	1.7	+2.2	−1.6	98.2
	25 ± 2 °C, ambient light	+5.3	6.0	+7.6	−3.2	94.0
LUTLNPs	4 °C, light-protected	+2.2	2.4	+2.7	−1.7	97.8
	25 ± 2 °C, ambient light	+6.7	7.6	+9.2	−3.1	93.4
CIPLUTLNPs	4 °C, light-protected	+1.4	1.9	+1.6	−1.5	98.4
	25 ± 2 °C, ambient light	+5.3	6.1	+7.0	−3.3	94.6
CCIPLUTLNPs	4 °C, light-protected	+1.5	2.3	+1.7	−1.8	98.2
	25 ± 2 °C, ambient light	+5.1	7.3	+6.2	−3.6	94.5

**Table 5:** Predicted ligand–protein interaction profiles of luteolin (LUT) and ciprofloxacin (CIP) across the multi-target panel—5α-Reductase, Androgen Receptor (AR), and human DNA topoisomerase IIα—derived from molecular docking analyses.

Target	Ligand	Ligand		Receptor			Interaction	Distance (Å)	E (kcal/mol)	Score
5α-Reductase	LUT	O	2	OD1	ASP	120 (A)	H-donor	2.78	−3.4	−5.106
		O	2	OD2	ASP	120 (A)	H-donor	3.42	−0.8	
		O	3	ND2	ASN	117 (A)	H-acceptor	3.26	−0.8	
	CIP	O	3	OE1	GLU	102 (A)	H-donor	2.87	−7.6	−5.345
Androgen Receptor	LUT	O	3	SD	MET	787 (A)	H-donor	3.39	−1.3	−7.002
		O	5	SD	MET	780 (A)	H-donor	3.55	−2.4	
		O	6	OD1	ASN	705 (A)	H-donor	2.95	−1.3	
	CIP	O	3	OD1	ASN	705 (A)	H-donor	2.67	−3.9	−7.86
		O	2	OG1	THR	877 (A)	H-acceptor	3.18	−0.8	
Human DNA Topoisomerase IIα	LUT	O	3	MG	MG	900 (A)	Metal	2.48	−1	−6.373
		6-ring		NZ	LYS	83 (A)	pi-cation	4.54	−0.6	
	CIP	O	2	MG	MG	900 (A)	Metal	2.15	−3	−7.692
		O	4	MG	MG	900 (A)	Metal	0.85	6425.6	
		6-ring		ND2	ASN	150 (A)	pi-H	4.48	−0.3	

## Data Availability

The original contributions presented in this study are included in the manuscript. Further inquiries can be directed to the corresponding author.
